# Olfactory Change Pattern After Endoscopic Sinus Surgery in Chronic Rhinosinusitis Patients

**DOI:** 10.7759/cureus.24597

**Published:** 2022-04-29

**Authors:** Abdullah Musleh, Ahmed S Al-Zomia, Ibarhim M Shahrani, Alwaleed Alshehri, Awad Alwadie, Fahad Alqhtani, Mosab Deajim, Sulafah Aljohani

**Affiliations:** 1 Otolaryngology - Head and Neck Surgery, King Khalid University Hospital, Khamis Mushait, SAU; 2 Medicine and Surgery, King Khalid University, Abha, SAU; 3 Medicine and Surgery, Taibah University, Madinah, SAU

**Keywords:** complications, nasal surgery, endoscopic surgery, olfactory dysfunction, chronic rhinosinusitis

## Abstract

Background

Chronic rhinosinusitis (CRS) is a chronic inflammation of the sinonasal mucosa that is clinically associated with sinus pressure, nasal congestion, rhinorrhea, and a decreased sense of smell that lasts more than 12 weeks. Endoscopic sinus surgery (ESS) for medically refractory CRS is mainly undergone to improve sinus function and access to topical medicinal treatments. However, olfactory changes after ESS can be unpredictable.

Aim

The current study aimed to assess olfactory change patterns after endoscopic sinus surgery in patients with chronic rhinosinusitis.

Methods

A record-based retrospective study was conducted in Aseer Central Hospital (ACH) ear, nose, and throat outpatient (ENT OPD) department and Khamis Mushayt General Hospital from August 15, 2021, to December 15, 2021. Data were collected using pre-structured data extraction sheet to avoid errors. Data extracted and collected included patients’ biodemographic data, CRS-associated symptoms, and endoscopic surgery-related data, including duration since surgery, presurgical medications, and duration of surgery. Also, postsurgical complications were extracted, especially olfactory complications.

Results

A total of 168 patients with chronic rhinosinusitis (CRS) and who had undergone endoscopic sinus surgery in the Aseer region were included. Patient ages ranged from 10 to 61 years, with a mean age of 39.8 ± 12.4 years old. Regarding the associated symptoms and complaints of the study patients, 110 (65.5%) complained of sinusitis, and 86 (51.2%) had allergic RS. The postsurgical complications of patients with CRS who had undergone endoscopic sinus surgery were olfactory dysfunction (OD) in 32 (19%), no complications in 115 (68.55%), and other nonspecific complications, such as headache, drowsiness, nose dryness, and bleeding, in 21 (12.55%). Also, 71 (42.3%) reported that they hardly perceive the fragrance in perfumeries.

Conclusion

In conclusion, olfactory impairment is a frequent clinical presentation in patients with CRS. In this study, olfactory dysfunction was improved, except among nearly one out of each five patients after ESS. Olfactory dysfunction was more among patients who had undergone recent surgery and those with chronic rhinosinusitis with nasal polyps (CRSwNP). Also, among patients who reported no complications, olfactory function did not return to normal in most patients as they hardly perceive fragrance.

## Introduction

Chronic rhinosinusitis (CRS) is a chronic inflammation of the sinonasal mucosa that is clinically associated with sinus pressure, nasal congestion, rhinorrhea, and a decreased sense of smell that lasts more than 12 weeks. CRS is classified into two major groups based on whether nasal polyps are present (chronic rhinosinusitis with nasal polyps (CRSwNP)) or absent (chronic rhinosinusitis without nasal polyps (CRSsNP)) [1].

Olfactory dysfunction (OD) among patients with chronic rhinosinusitis (CRS) is frequent, with prevalence varying from 48% to 83% based on how olfactory dysfunction is demarcated [1-4]. Medical treatment improves olfactory dysfunction in CRS, although failed medical therapy for CRS necessitates undergoing endoscopic sinus surgery (ESS) [5]. It is known that surgical intervention includes removing obstructing polyps and mucosa, restoring normal airflow, and improving the inflammatory response, which all should restore olfactory function [6].

In spite of the high prevalence of olfactory dysfunction in CRS patients with its negative impact on the quality of life (QOL) and disease severity, olfaction is understudied within the larger rhinosinusitis population [7]. Worldwide, CRS is the main cause of olfactory dysfunction detected nearly in one of every eight adults in the United States [4]. Annually, more than 250,000 persons undergo endoscopic sinus surgery for CRS in the United States [8]. Recently, the literature reported that the majority of patients may report symptomatic improvement after endoscopic sinus surgery, which may scatter over time [9,10]. Most of these studies are based mainly on retrospective approaches, subjective meanings of patient “improvement” after surgery, secondary analyses, and/or a five-year maximum time frame for prospectively collected long-term follow-up results [11,12].

Endoscopic sinus surgery (ESS) for medically refractory CRS is mainly undergone to improve sinus function and access to topical medicinal treatments. However, olfactory changes after ESS can be unpredictable [13]. Although it is expected that olfactory dysfunction in chronic sinusitis will be back to normal after surgical treatment, with endoscopic sinus surgery and long-term medical therapy, a significant number of patients remain anosmic. Several publications have recognized clinically this type of olfactory impairment. However, the pathophysiology of this disease is yet unknown [14]. The current study aimed to assess olfactory change patterns after endoscopic sinus surgery in patients with chronic rhinosinusitis using the Self-reported Mini Olfactory Questionnaire.

## Materials and methods

A record-based retrospective study was conducted in Aseer Central Hospital (ACH) ear, nose, and throat outpatient (ENT OPD) department and Khamis Mushayt General Hospital from August 15, 2021, to December 15, 2021. Data were collected using pre-structured data extraction sheet to avoid errors. Adult and adolescent patients with chronic rhinosinusitis who had undergone endoscopic sinus surgery were included. Adult or adolescent patients with olfactory changes not secondary to CRS and others with CRS who have not undergone endoscopic sinus surgery were excluded. Data extracted and collected included the patients’ biodemographic data, CRS-associated symptoms, and endoscopic surgery-related data, including duration since surgery, presurgical medications, and duration of surgery. Also, postsurgical complications were extracted, especially olfactory complications.

Data analysis

After data were extracted, it was revised, coded, and input into the statistical software SPSS version 22 (IBM Corp., Armonk, NY, USA). All statistical analyses were done using two-tailed tests. A p-value of less than 0.05 was statistically significant. Descriptive analysis based on the frequency and percent distribution was done for all variables, including the patients’ sociodemographic data, medical history, ENT-related symptoms, duration of having CRS, medications, surgery-related data, and postsurgical complications, including olfactory dysfunction. The distribution of patients’ postsurgical complications by their bio-clinical data and surgery history was tested using the Pearson chi-square test and the exact probability test for small frequency distributions.

## Results

A total of 168 patients with chronic rhinosinusitis (CRS) and who had undergone endoscopic sinus surgery in the Aseer region were included. Patient ages ranged from 10 to 61 years, with a mean age of 39.8 ± 12.4 years old. A total of 95 (56.5%) patients are males, and 120 (71.4%) were working or students. As for education, 23 (13.75%) had below the secondary level of education, and 108 (64.3%) had a university level of education or above. Additionally, 131 (78%) were married, and 160 (95.25%) were Saudi. Smoking or heavy exposure to smoke was reported in 29 (17.3%), and only two (1.2%) were alcohol users. A total of 25 (14.9%) patients were diabetic, 11 (6.55%) had depressive symptoms, and 111 (66.1%) had no other comorbidities (Table 1).

**Table 1:** Biodemographic data of patients with chronic rhinosinusitis in the Aseer region of Saudi Arabia

Biodemographic data	Number	%
Age in years		
<30	32	19%
30-39	54	32.1%
40-49	56	33.3%
50+	26	15.5%
Gender		
Male	95	56.5%
Female	73	43.5%
Work		
Not working	48	28.6%
Working	120	71.4%
Education		
Below secondary	23	13.7%
Secondary	37	22%
University/above	108	64.3%
Marital status		
Single	29	17.3%
Married	131	78%
Divorced/widow	8	4.8%
Nationality		
Saudi	160	95.2%
Non-Saudi	8	4.8%
Smoker or heavily exposed to smoke		
Yes	29	17.3%
No	139	82.7%
Are you an alcohol user (currently)?		
Yes	2	1.2%
No	166	98.8%
Other comorbidities		
None	111	66.1%
DM	25	14.9%
HTN	8	4.8%
Depression disorder	11	6.5%
Others	13	7.7%

**Figure 1:**
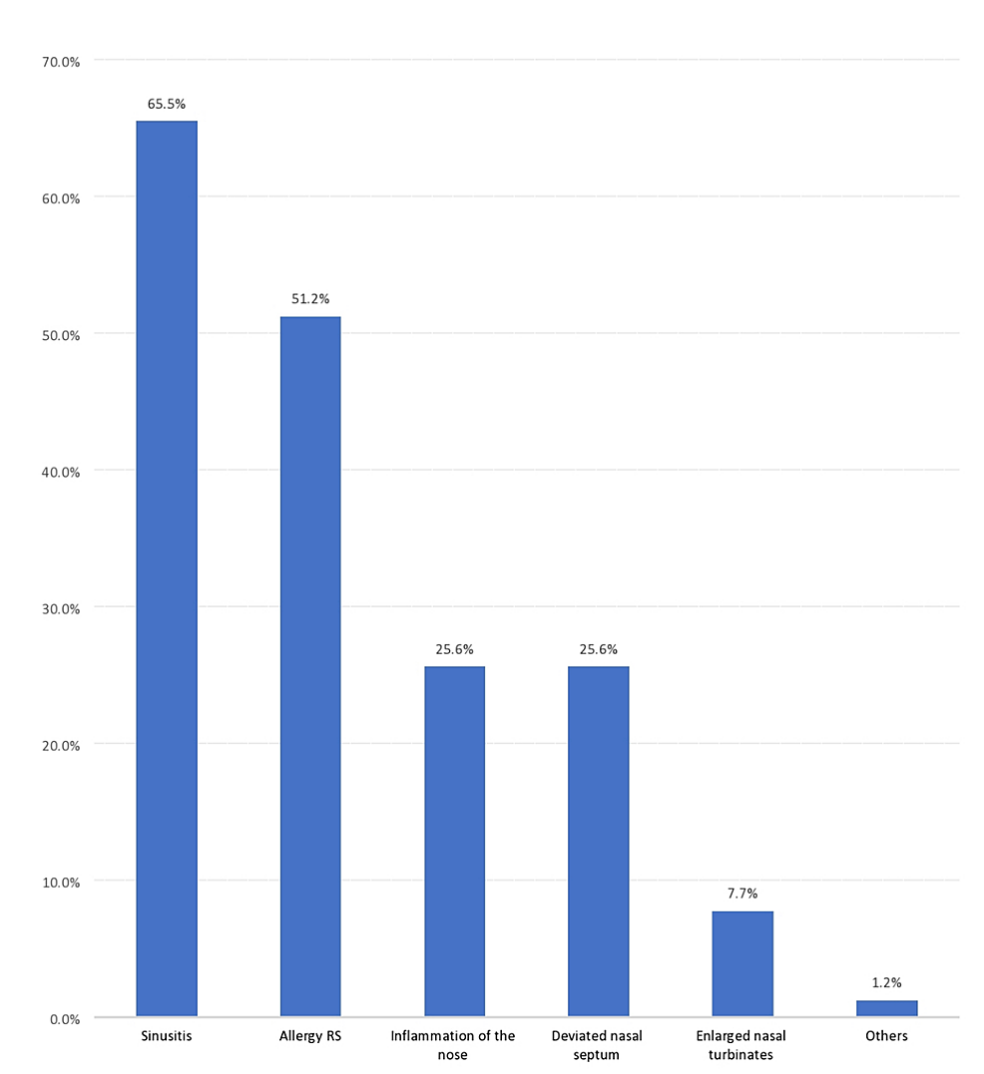
Associated symptoms and complaints among the study patients

A total of 110 (65.5%) complained of sinusitis, 86 (51.2%) had allergic RS, 43 (25.6%) complained of inflammation of the nose, 43 (25.6%) had deviated nasal septum, and 13 (7.7%) complained of enlarged nasal turbinates (Figure 1).

A total of 104 (61.9%) patients complained of CRS for more than one year, 81 (48.2%) were diagnosed with nasal polyposis (CRSwNP), and 78 (46.4%) had been diagnosed with asthma or allergy. Steroid intake was reported in 35 (20.8%) patients. As for surgery, 11 patients (6.5%) underwent endoscopic sinus surgery six months ago, 31 (18.5%) underwent surgery for 1-5 years, and 121 (72%) underwent surgery for more than five years (Table 2).

**Table 2:** Clinical and surgery data regarding CRS among the study patients

Clinical and surgery data	Number	%
Duration of RS		
<1 year	64	38.1%
>1 year	104	61.9%
Have you ever been diagnosed with nasal polyposis (CRSwNP)?		
Yes	81	48.2%
No	87	51.8%
Have you ever been diagnosed with asthma or allergy?		
Yes	78	46.4%
No	90	53.6%
Do you depend on oral steroids?		
Yes	35	20.8%
No	133	79.2%
Duration since endoscopic sinus surgery		
<6 months	11	6.5%
6 months-1 year	5	3%
1-5 years	31	18.5%
> 5 years	121	72%

Regarding postsurgical complications, 32 (19%) patients experienced olfactory dysfunction after endoscopic sinus surgery, 115 (68.55%) had no complications, and 21 (12.55%) had other nonspecific complications, such as headache, drowsiness, nose dryness, and bleeding. Also, 71 (42.3%) reported that they hardly perceive the fragrance in perfumeries.

**Table 3:** Postsurgical complications among the patients with CRS who had undergone endoscopic sinus surgery

Complications	Number	%
Postsurgical complications		
None	115	68.5%
Loss of olfactory function	32	19%
Others	21	12.5%
Hardly perceive the fragrance in perfumeries		
Yes	71	42.3%
No	97	57.7%

Olfactory dysfunction was detected among 23.2% of male patients compared to 13.7% of females (p = 0.049). Also, 24% of patients with CRS for more than one year experienced postsurgical olfactory dysfunction versus 10.9% of those who had the disease for less than one year (p = 0.020). Postsurgical olfactory dysfunction was reported among 29.6% of patients who had CRSwNP in comparison to 9.2% of others without (p = 0.002). Also, 37.1% of the patients who received steroids had postsurgical olfactory dysfunction versus 14.3% of others who did not (p = 0.007). Additionally, postsurgical olfactory dysfunction was detected among 40% of patients who had undergone surgery for six months to one year compared to 14.9% of those who underwent the surgery for more than five years (p = 0.005) (Table 4).

**Table 4:** Distribution of postsurgical olfactory dysfunction by patient bio-clinical data P: Pearson X2 test, $: exact probability test, *: p < 0.05 (significant)

Factors	Postsurgical complications						p-value
	None		Loss of olfactory function		Others		
	Number	%	Number	%	Number	%	
Age in years							0.565^$^
<30	21	65.6%	6	18.8%	5	15.6%	
30-39	34	63%	14	25.9%	6	11.1%	
40-49	43	76.8%	6	10.7%	7	12.5%	
50+	17	65.4%	6	23.1%	3	11.5%	
Gender							0.049*
Male	58	61.1%	22	23.2%	15	15.8%	
Female	57	78.1%	10	13.7%	6	8.2%	
Work							0.330
Not working	32	66.7%	12	25%	4	8.3%	
Working	83	69.2%	20	16.7%	17	14.2%	
Education							0.171^$^
Below secondary	15	65.2%	3	13%	5	21.7%	
Secondary	21	56.8%	9	24.3%	7	18.9%	
University/above	79	73.1%	20	18.5%	9	8.3%	
Smoker or heavily exposed to smoke							0.349
Yes	21	72.4%	3	10.3%	5	17.2%	
No	94	67.6%	29	20.9%	16	11.5%	
Are you an alcohol user (currently)?							0.251^$^
Yes	1	50%	0	0%	1	50%	
No	114	68.7%	32	19.3%	20	12%	
Other comorbidities							0.628^$^
None	75	67.6%	21	18.9%	15	13.5%	
DM	18	72%	5	20%	2	8%	
HTN	6	75%	1	12.5%	1	12.5%	
Depression disorder	5	45.5%	3	27.3%	3	27.3%	
Others	11	84.6%	2	15.4%	0	0%	
Duration of RS							0.020*
<1 year	52	81.3%	7	10.9%	5	7.8%	
>1 year	63	60.6%	25	24%	16	15.4%	
Have you ever been diagnosed with nasal polyposis (CRSwNP)?							0.002*
Yes	46	56.8%	24	29.6%	11	13.6%	
No	69	79.3%	8	9.2%	10	11.5%	
Have you ever been diagnosed with asthma or allergy?							0.437
Yes	50	64.1%	18	23.1%	10	12.8%	
No	65	72.2%	14	15.6%	11	12.2%	
Do you depend on oral steroids?							0.007*
Yes	20	57.1%	13	37.1%	2	5.7%	
No	95	71.4%	19	14.3%	19	14.3%	
Duration since endoscopic sinus surgery							0.005*^$^
<6 months	6	54.5%	3	27.3%	2	18.2%	
6 months-1 year	3	60%	2	40%	0	0%	
1-5 years	13	41.9%	9	29%	9	29%	
>5 years	93	76.9%	18	14.9%	10	8.3%	

## Discussion

The current study aimed to assess olfactory dysfunction after endoscopic sinus surgery among patients with CRS in the Aseer region of Saudi Arabia.

Olfactory dysfunction (OD) in CRS is mainly due to mucosal inflammation, which can harm olfaction by physically obstructing airflow and odorant transfer to an otherwise unaffected olfactory epithelium, whether it induces mucosal swelling or polyp formation [15]. On the other hand, inflammation-mediated damage to the olfactory epithelium may affect olfaction directly [16].

The study showed that two-thirds of the patients who had undergone ESS had no postsurgical complications such as loss of olfactory function, visual loss, spinal fluid leak, and bleeding.

Loss of olfactory function was reported postoperatively among about one-fifth of the patients (19%), which means that one out of each five has olfactory dysfunction. Also, a bit less than half of the patients (42.3%) hardly perceive the fragrance of perfumeries. Olfactory dysfunction was significantly higher among male patients, those with CRS for more than one year due to chronically inflamed mucosa, and patients with nasal polyps because inflamed mucosa obstructs the sinuses’ capacity to drain and nasal polyps obstruct the nasal channel; both of these conditions contribute to a loss of smell. Also, patients who were on steroids before surgery experienced postsurgical olfactory dysfunction and others with recent surgery.

The literature supporting olfactory enhancement after ESS is contradictory. Improvements reported range from 25% to 100%, with others reporting no change or even dysfunction [17-21]. This inconsistency may be a result of variabilities in olfactory outcomes, subjective versus objective assessment, study population, definitions of improvement, duration of patient’s follow-up, or preoperative olfactory status. As such, advising patients on postoperative smell recovery is increasingly difficult. Kohli et al. [22] reported that olfactory measures among patients after endoscopic sinus surgery showed significant improvement in mixed CRS patients (those with and without polyps). Chronic rhinosinusitis mixed patients demonstrated nonsignificant improvements via Sniffin’ Sticks threshold and Brief Smell Identification Test. When separated, polyp patients and dysosmic patients experienced the highest levels of olfactory improvement. Polyp patients improved by 7.87 on the 40-item Smell Identification Test. Also, Delank et al. [2] found that 80% of the hyposmic patients complained of an isolated reduction of their ability to discriminate odors. Postoperative improvements were reported in 70%. About 25% achieved normosmia postoperatively among the hyposmic patients, but only in 5% of the anosmic patients. Mohanty [23] estimated that olfactory scores in anosmic patients significantly improved after ESS at a three-month follow-up. Only a few hyposmic patients improved after surgery, and others did not show any change. Among normosmic patients, 80% showed no change after surgery, whereas 20% became hyposmic postoperatively. None of the normosmic patients became anosmic after surgery.

Finally, we obtained the patient data from two major hospitals in the Aseer region, and due to the difficulties in contacting patients, we had an insufficient sample size that does not represent the whole population of the Aseer region. We recommend more research be done in this field to achieve the optimal sample size that will represent the whole population of the Aseer region.

## Conclusions

In conclusion, olfactory impairment is a frequent clinical presentation in patients with CRS. In this study, olfactory dysfunction was improved, except among nearly one out of each five patients after ESS. Olfactory dysfunction was more among patients who had undergone recent surgery and those with CRSwNP. Also, among patients who reported no complications, olfactory function did not return to normal in most patients as they hardly perceive fragrance. Olfactory impairment is important patient safety and quality of life issue for patients with CRS and one that requires continued research. Large-scale studies are recommended taking into consideration the initial complaint, medical conditions, duration of follow-up, and severity of CRS to precisely assess post-endoscopic surgery olfactory dysfunctions.
